# Changes in Birth Weight between 2002 and 2012 in Guangzhou, China

**DOI:** 10.1371/journal.pone.0115703

**Published:** 2014-12-22

**Authors:** Yong Guo, Yu Liu, Jian-Rong He, Xiao-Yan Xia, Wei-Jian Mo, Ping Wang, Qiong Feng, Charles P. Larson, Hui-Min Xia, Xiu Qiu

**Affiliations:** 1 Division of Birth Cohort Study, Guangzhou Women and Children's Medical Center, Guangzhou, China; 2 Department of Health Care, Guangzhou Women and Children's Medical Center, Guangzhou, China; 3 Guangzhou Women and Children's Health Information Center, Guangzhou, China; 4 Centre for International Child Health, BC Children's Hospital and University of British Columbia, Vancouver, Canada; Institute for Health & the Environment, United States of America

## Abstract

**Background:**

Recent surveillance data suggest that mean birth weight has begun to decline in several developed countries. The aim of this study is to examine the changes in birth weight among singleton live births from 2002 to 2012 in Guangzhou, one of the most rapidly developed cities in China.

**Methods:**

We used data from the Guangzhou Perinatal Health Care and Delivery Surveillance System for 34108 and 54575 singleton live births with 28–41 weeks of gestation, who were born to local mothers, in 2002 and 2012, respectively. The trends in birth weight, small (SGA) and large (LGA) for gestational age and gestational length were explored in the overall population and gestational age subgroups.

**Results:**

The mean birth weight decreased from 3162 g in 2002 to 3137 g in 2012 (crude mean difference, −25 g; 95% CI, −30 to −19). The adjusted change in mean birth weight appeared to be slight (−6 g from 2002 to 2012) after controlling for maternal age, gestational age, educational level, parity, newborn's gender and delivery mode. The percentages of SGA and LGA in 2012 were 0.6% and 1.5% lower than those in 2002, respectively. The mean gestational age dropped from 39.2 weeks in 2002 to 38.9 weeks in 2012. In the stratified analysis, we observed the changes in birth weight differed among gestational age groups. The mean birth weight decreased among very preterm births (28–31 weeks), while remained relatively stable among other gestational age subcategories.

**Conclusions:**

Among local population in Guangzhou from 2002 to 2012, birth weight appeared to slightly decrease. The percentage of SGA and LGA also simultaneously dropped, indicating that newborns might gain a healthier weight for gestational age.

## Introduction

Birth weight is an important predictor of both short and long-term child health outcomes. Low or high birth weight is associated with higher risks for neonatal morbidity and mortality [1]. Because of the improvement in socioeconomic conditions and prenatal care, the average birth weight has increased in many countries during the last three decades [2]–[4], including the United States, Canada, the UK, Japan and China. However, more recent surveillance data suggest that mean birth weight has begun to decline in some developed countries. In 2005, compared with 1990, mean birth weight decreased from 3441 g to 3389 g among term singleton neonates in the United States [5]. Similar trends have also been observed in France and Germany [6], [7].

In recent years, a few studies examined secular trends of birth weight in China. Data from Perinatal Health Care Surveillance System in southeast China showed the mean birth weight for all term infants increased from 3296 g in 1994 to 3378 g in 2000, then leveled off to 3369 g in 2005 [2]. Another retrospective investigation in Henan province, reported decrease in mean birth weight from 1987 to 2006 [8]. However, these studies were not all population based, or limited to term infants.

Guangzhou is one of the most developed regions in China. During the last few decades, there has been a dramatic change in the economic conditions and the number of births per year in Guangzhou. Demographic, social and economic conditions are known as determinants of health in general, including birth weight [9], [10]. In order to develop more effective prenatal care intervention to improve both mothers' and children's health, better understanding of changes in birth weight in Guangzhou population is needed.

In Guangzhou Annual Maternal and Child Health Report, we found that the mean birth weight tended to decrease linearly from 2002 to 2012 [11]. Therefore, in the present study we used the data of the first year (2002) and the last year (2012) to further assess the characteristics of changes in birth weight in Guangzhou.

## Methods

### Data source and study population

The electronic Guangzhou Perinatal Health Care and Delivery Surveillance System (GPHCDSS) database was implemented in 2000, and covers over 97% of deliveries in Guangzhou [12]. Within the surveillance system, birth information of neonates born in hospitals is reported to Guangzhou Municipal Health Bureau via computer network and is used to issue birth certificate. For each hospital, one trained health worker was responsible for registering the information of births. The data was confirmed by the Chief of Midwife in the hospital and the Chief of Physician. The Department of Medical Administration and the newborn's parent also validated the information when the birth certificate was issued. Guangzhou Municipal Health Bureau verifies the GPHCDSS data annually through sampling survey. Thus the information from the GPHCDSS is regarded as having high reliability. From this surveillance system information on maternal demographics and delivery summary were collected. Registered midwives routinely measure birth weight using an electronic weighing scale within half an hour of delivery. Gestational age at birth was based on an ultrasound examination in the first or second trimester and was expressed as completed weeks. When the ultrasound examination was unavailable, the last menstrual period was used to calculate gestational age.

The analyses limited to singleton live births among local population delivered between 28 and 41 completed weeks of gestation in 2002 and 2012. The following data were extracted from the electronic GPHCDSS records: maternal age and educational level, parity, delivery mode, date of birth, newborn gender, gestational age and birth weight. A total of 88822 records of singleton live births (28–41 completed weeks of gestation) born in 2002 and 2012 were retrieved and 139 were excluded due to misclassification of gestational age or invalid birth weight. Final analyses included 88683 singleton births (34108 births in 2002 and 54575 births in 2012). Of the total, 47420 were delivered vaginally.

### Classifications

Newborns were categorized according to gestational age in completed weeks as: very preterm birth (28–31 weeks), moderate or late preterm birth (32–36 weeks) [13], early term birth (37–38 weeks), full term birth (39–40 weeks) and late term birth (41 weeks) [14], [15]. Birth weight was classified into very low birth weight (<1500 g), low birth weight (1500–2499 g), normal birth weight (2500–3999 g), and macrosomia (≥4000 g). Small for gestational age (SGA) was defined as fetal growth less than the 10th percentile at each completed week of gestation, and defined large for gestational age (LGA) as greater than 90th percentile, according to reference data based on all Guangzhou live births in 2009–2011 [12]. Delivery modes were divided into vaginal delivery, cesarean delivery and assisted breech delivery.

The institutional review board of Guangzhou Women and Children's Medical Center approved the study.

### Statistical analyses

To avoid misclassification of gestational age and invalid birth weight that might bring bias in gestational age-specific birth weight distributions [16], we first detected and eliminated these implausible values before analyses. Because of bimodal distributions of birth weight for 28–34 weeks of gestation and symmetric unimodal distributions for later gestational ages, we used different methods to identify erroneous values. As shown by other studies, a Gaussian mixture model with two components was effective to identify misclassification of early gestational age and here we followed Marcelo et al's procedure and SAS codes [16]. Specifically, it is hypothesized that at gestational age x, the birth weight y arise from two normal components: *f_p_*, the primary component which describes the actual weight distribution, and *f_s_*, the secondary component, which consisted of implausible values. That can be briefly expressed in a formula as: 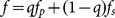. Whether a given observation (*x*, *y*) belongs to the *f_p_* distribution depends on the weight estimation: *w =  qf_p_/f*. The birth weight values that have a lower probability of belonging to primary distribution than 0.5 were considered implausible [17], [18].

At each of the weeks of gestational age 35 to 41, where birth weight distributions were symmetric unimodal with long tails, robust regression was used to do M estimation to identify invalid birth weights [19]. Iteractively reweighted least square (IRLS) procedure was performed in the procedure and birth weights with large residuals exceeding 3.89 SD of the residuals would be deemed to be extreme and so were then removed from the data as invalid.

We performed multiple linear regression analysis to examine the birth weight changes after adjustment for maternal age, gestational age, educational level, parity, newborn's gender and delivery mode. To examine the impact of maternal and newborn's characteristics changes over time on birth weight for gestational age, we standardized the proportions of SGA and LGA in 2012 to the population in 2002 by maternal age, educational level, parity, delivery mode and gestational age distributions (direct standardization).

Statistical analyses were performed using SAS statistical software version 9.2 (SAS Institute, Cary, NC).

## Results

Over the 10-year period, the number of births per year increased by 60.0% in Guangzhou. Table 1 shows information on maternal and newborn characteristics for the years 2002 and 2012. The mean maternal age increased from 27.1±4.0 years to 27.5±4.4 years. An increasing percentage of neonates were born to mothers with age more than 35 years and higher parity. There were no marked temporal trends in neonatal sex (54.3% males in 2002 and 54.7% males in 2012). From 2002 to 2012, the gestational age distribution shifted towards shorter, with a mean decrease of 0.3 weeks. The rate of preterm increased from 5.1% to 6.2%. When the modes of delivery are compared cesarean delivery and assisted breech delivery decreased, respectively, from 47.1% and 2.6% in 2002 to 43.5% and 1.1% in 2012.

**Table 1:** Maternal and newborn characteristics among singleton live births in 2002 and 2012.

	2002	2012
	(n = 34108)	(n = 54575)
Maternal characteristics		
Age (yrs)	27.1±4.0	27.5±4.4
Less than 20	238 (0.7)	600 (1.1)
20–24	8823 (25.9)	12927 (23.7)
25–29	16965 (49.7)	25417 (46.6)
30–34	6575 (19.3)	11563 (21.2)
35 or above	1465 (4.3)	4036 (7.4)
Missing	42 (0.1)	32 (0.1)
Education		
Elementary school or less	1772 (5.2)	436 (0.8)
Junior school	13221 (38.8)	11178 (20.5)
High school	13289 (39.0)	33426 (61.2)
College	4362 (12.8)	5071 (9.3)
Undergraduate or above	1431 (4.2)	4417 (8.1)
Missing	33 (0.1)	47 (0.1)
Parity		
1	27898 (81.8)	39509 (72.4)
2 or more	6207 (18.2)	15061 (27.6)
Missing	3 (0.0)	5 (0.0)
Newborn characteristics		
Gender		
Male	18521 (54.3)	29853 (54.7)
Female	15587 (45.7)	24722 (45.3)
Mean gestational age (weeks)	39.2±1.5	38.9±1.4
28–31	102 (0.3)	164 (0.3)
32–36	1641 (4.8)	3220 (5.9)
37–38	10618 (31.1)	20629 (37.8)
39–40	18565 (54.4)	28270 (51.8)
41	3182 (9.3)	2292 (4.2)
Mean birth weight (g)	3162±430	3137±421
Birth weight for gestational age		
Small for gestational age	3154 (9.2)	4677 (8.6)
Large for gestational age	3396 (10)	4663 (8.5)
Birth weight classifications		
Very low birth weight	78 (0.2)	115 (0.2)
Low birth weight	1424 (4.2)	2650 (4.9)
Normal birth weight	31530 (92.4)	50529 (92.6)
Macrosomia	1076 (3.2)	1281 (2.3)
Delivery modes		
Vaginal delivery	17136 (50.2)	30284 (55.5)
Cesarean delivery	16072 (47.1)	23717 (43.5)
Asssisted breech delivery	899 (2.6)	574 (1.1)
Missing	1 (0.0)	0 (0.0)

Data are expressed as mean ± standard deviation or n(%).

**Figure 1:**
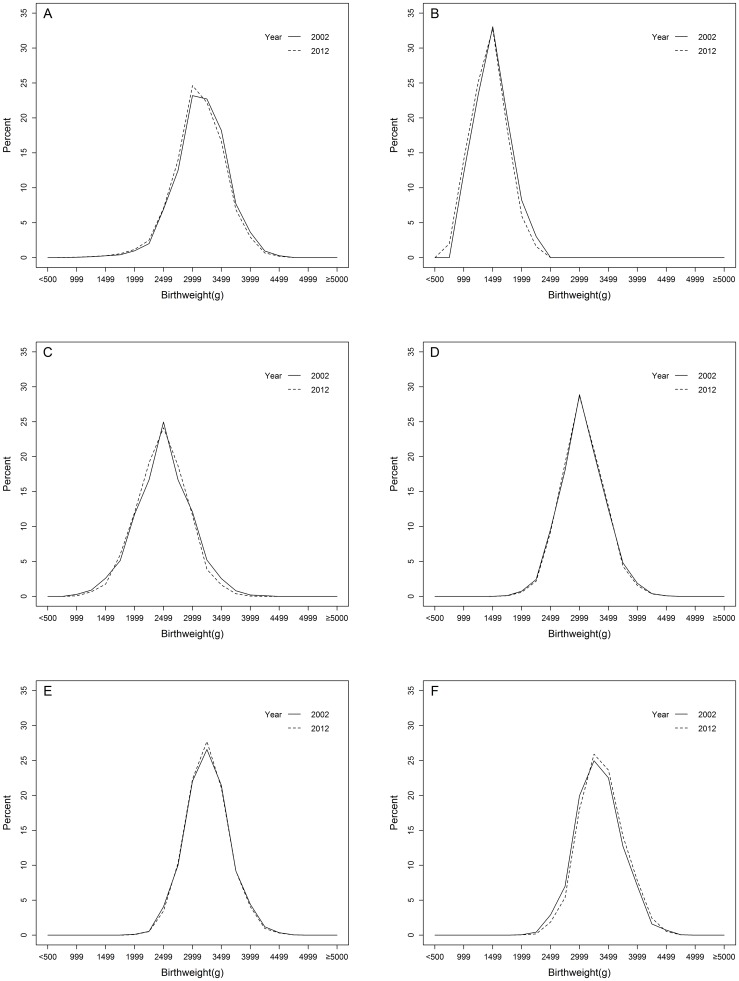
Percentage distribution of singleton live births by birthweight, born in 2002 and 2012. (A, 28–41 completed weeks of gestation; B, 28–31 completed weeks of gestation; C, 32–36 completed weeks of gestation; D, 37–38 completed weeks of gestation; E, 39–40 completed weeks of gestation; F, 41 completed weeks of gestation).

There were no significant tendencies for a systematic change in birth weight within different completed weeks of gestation. Fig. 1 A–F represented the percentage distribution of singleton live births by birth weight, born in 2002 and 2012. For the overall birth weight, the curve of 2012 was shifted to the left relative to that of 2002 (Fig. 1 A) and the shapes of birth weight distributions were similar, indicating birth weight decreased from 2002 to 2012. Gestational-age specific birth weight comparison showed that the 2012 curves shifted leftward at 28–31 weeks (Fig. 1 B) and 32–36 weeks (Fig. 1 C), almost coincided at 37–38 weeks and 39–40 weeks (Fig. 1 D, E) and shifted rightward at 41 weeks (Fig. 1 F), suggesting birth weight decreased among preterm births (<37 weeks), remained stable among early term births (37–38 weeks) and full term births (39–40 weeks), and increased among late term births (41 weeks) from 2002 to 2012, respectively. The crude mean birth weight among all singleton live births decreased by 25 g (95% CI: −30 to −19) (Table 2). After adjustment for maternal characteristics, mean birth weight decreased by 6 g among all singleton live births from 2002 to 2012 (Table 2). In the subgroup analysis, the mean birth weight decreased among very preterm births (28–31 weeks), while remained relatively stable among other gestational age subcategories after adjustment for maternal characteristics (Table 2).

**Table 2:** Changes in birth weight among singleton live births between 2002 and 2012.

	2002	2012	Unadjusted change from 2002 to 2012 (95%CI)	Adjusted change from 2002 to 2012 (95%CI)
All live births	3162±430	3137±421	−25 (−30 to −19)	−6 (−11 to 0)*
28–31 weeks	1501±317	1472±313	−29 (−53 to −5)	−24 (−46 to −2)**
32–36 weeks	2558±469	2540±430	−18 (−34 to −2)	−11 (−28 to 6)**
37-38 weeks	3071±380	3066±367	−5 (−14 to 3)	−8 (−18 to 2)**
39–40 weeks	3247±385	3249±368	2 (−4 to 9)	−6 (−13 to 1)**
41 weeks	3344±383	3366±363	22 (3 to 42)	8 (−10 to 26)**

Data are expressed as mean ± standard deviation.

^*^ Adjusted for maternal age, gestational age, educational level, parity, newborn's gender and delivery mode.

^**^ Adjusted for maternal age, educational level, parity, newborn's gender and delivery mode.

In the overall singleton live births, the proportion of very low birth weight remained fairly stable at 0.2%, while low birth weight increased from 4.2% in 2002 to 4.9% in 2012. Macrosomia decreased from 3.2% to 2.3%. The percentage of SGA and LGA simultaneously dropped, respectively, by 0.6% and 1.5% (Table 1). Table 3 showed the changes of SGA and LGA proportion stratified by maternal and newborn characteristics (Table 3). After standardized to maternal educational level structure in 2002, the proportion of SGA in 2012 (9.2%) was close to that in 2002 (9.2%) (Table 3). Standardizations to other maternal characteristics in 2002 did not substantially change the proportions of SGA and LGA in 2012 (Table 3).

**Table 3:** Percentage of singleton live births with SGA, AGA and LGA, by maternal and newborn characteristics in 2002 and 2012.

	2002			2012		
	SGA	AGA	LGA	SGA	AGA	LGA
Overall	9.2	80.8	10.0	8.6	82.9	8.5
Maternal age (yrs)						
Less than 20	10.6	83.2	6.2	13.0	80.6	6.4
20–24	10.4	82.0	7.6	9.9	83.4	6.8
25–29	8.9	80.8	10.3	8.1	83.4	8.5
30–34	8.5	79.8	11.7	8.0	82.5	9.5
35 or above	9.9	76.5	13.6	8.1	79.7	12.2
Standardized maternal age*	9.2	80.8	10.0	8.6	83.0	8.4
Maternal education						
Elementary school or less	13.4	78.4	8.2	13.3	80.5	6.2
Junior school	11.1	80.7	8.3	10.3	82.3	7.5
High school	8.6	80.9	10.6	8.4	82.9	8.7
College	5.7	81.6	12.7	7.5	83.7	8.8
Undergraduate or above	4.5	81.7	13.8	6.0	83.7	10.2
Standardized maternal education*	9.2	80.8	10.0	9.2	82.7	8.2
Parity						
1	9.3	80.8	9.9	8.6	82.9	8.5
2 or more	9.0	81.0	10.0	8.4	82.9	8.7
Standardized parity*	9.2	80.8	10.0	8.6	82.9	8.5
Newborn gender						
Male	9.1	81.2	9.7	8.5	83.1	8.4
Female	9.4	80.3	10.3	8.7	82.6	8.7
Standardized newborn gender*	9.2	80.8	10.0	8.6	82.9	8.5
Gestational age (weeks)						
28–31	6.2	76.0	17.8	9.6	72.9	17.5
32–36	12.7	77.3	10.0	9.8	83.8	6.4
37–38	9.0	81.6	9.4	8.9	83.3	7.8
39–40	9.5	80.5	10.0	8.4	82.5	9.1
41	7.0	82.4	10.6	5.8	83.4	10.9
Standardized gestational age*	9.2	80.8	10.0	8.4	82.9	8.7

SGA, small for gestational age; AGA, appropriate size for gestational age; LGA, large for gestational age.

^*^ Standardized to the 2002.

## Discussion

The present study analyzing a large sample size based on the local population in Guangzhou, China has shown that birth weight had a slight decrease and the shifts in birth weights between 2002 and 2012 were inconsistent among gestational age groups. The crude birth weight decreased among singleton preterm births, while remained relatively stable among early term (37–38 weeks) and full term (39–40 weeks) births and increased among late term (41 weeks) births. Results of adjusted regression analyses showed that the decreasing birth weight change was limited to very preterm birth (28–31 weeks), while birth weight among other gestational age subcategories remained relatively stable.

This finding of changes in birth weight is not consistent with that found in previous studies investigating birth weight trends. Most of previous studies included only births that occurred before 2005 and the analyses were restricted to full term births. On analysis of term singleton births in the United States from 1990 to 2005 observed decreases in birth weight (−52 g in the overall population, −79 g in a homogenous low-risk subgroup) and the mean birth weight declined within each completed week of gestational age [5]. In another analysis from southeast China, the increments of birth weight differed by gestational age and rose the most at 38–41 weeks (more than 80 g) from 1994 to 2005 [2]. In present study, we observed the birth weight among very preterm birth (28–31 weeks) decreased. One of the possible explanations might be due to the improvement of obstetric technologies. More preterm babies with poor growth, who had a high risk of in utero death in the past, could survive with advanced supportive care nowadays. This could be a contributor to birth weight decrease for preterm births because our analysis was restricted to live birth.

Weight for gestational age is a better outcome measure accounting fetal growth [20]. In our study, both SGA and LGA births decreased from 2002 to 2012. The percentage changes of weight for gestational age were partly attributed to changes in maternal socio-demographic characteristics [21]. It was reported that SGA was more prevalent among mothers with low levels of education [22]. In present study, we observed the proportion of mothers who had education level below high school decreased, which might be one of factors leading to the declined SGA proportion. The declining trend of LGA proportion remained after standardization for maternal age, education, parity and gestational age, suggesting that these factors could not account for observed trend. Several other factors might contribute to decreased proportion of LGA. Enhanced social support, health education, clinical services and nutritional counseling that maintain optimized gestational weight gain might resulted in lower prevalence of LGA newborns [23]. In addition, the new diagnostic thresholds for gestational diabetes mellitus tripled the prevalence [24]. Women diagnosed with gestational diabetes mellitus likely to receive interventions including glucose monitoring, obstetric monitoring and clinical care, resulting in improved pregnancy outcomes and appropriate birth weight.

The changes in gestational age and birth weight for gestational age might be affected more strongly among preterm than term births due to changes over time in the assessment of gestational age [5]. Over time, if smaller newborns were increasingly likely to be classified as preterm rather than term, then we would see an upward trend in birth weight at term, but the weight for gestational age among neonates born before term might not change or decrease [5]. This may be one of the reasons for different shifts in birth weight between preterm and term observed in the present study. The method for estimating gestational age could also influence perinatal outcomes [25]. We could not gather precise information of the method used to assess gestational age in individual case. Whether gestational ages were corrected by ultrasound or not were not recorded in our surveillance system. Hence, we could not differentiate between those cases evaluated with one method or the other, though the use of ultrasound to estimate gestational age had increased in Guangzhou. Obstetric estimate of gestational age from the birth certificate had been validated for the surveillance in one study [26].

The principal limitation of our study was lack of more precise information to answer more specific questions that were addressed in other studies from general population [27], [28]. The maternal characteristics that might contribute to birth weight such as changes in pre-pregnancy BMI, gestational weight gain, socioeconomic factors, maternal diet and medical conditions [5], [29] were not recorded in birth records of GPHCDSS. We were unable to demonstrate the effects of these factors on birth weight. But the major strength of our study was first to use a surveillance data that provides a large representative population size to examine the changes in birth weight in Guangzhou over time. The study results have several implications. Firstly, the percentage of SGA and LGA decreased from 2002 to 2012, suggesting that newborns might gain a healthier weight. Secondly, the overall birth weight did not change substantially from 2002 to 2012 in the present study, while several recent studies found the prevalence of overweight and obesity was increasing among children and adolescents in the same city [30], [31]. Thus, we speculated overnutrition after birth might have more significant impact on childhood obesity than birth weight. This focus could help to guide the formulation of basic hypotheses for future research.

## Conclusions

We analyzed the changes in birth weight between 2002 and 2012 in singleton live births in a large population in Guangzhou. There was a slight decrease in mean birth weight. The percentage of SGA and LGA also simultaneously dropped, indicating that newborns might gain a healthier weight for gestational age.

## Supporting Information

S1 Fig **Frequency distributions of birth weight for 28-33 weeks of gestation before (upper) and after (lower) excluding the implausible data in 2002.** The upper one shows the frequency distributions of birth weight before excluding. The lower one shows the frequency distributions of birth weight after excluding.
(TIF)

S2 Fig **Frequency distributions of birth weight for 28-33 weeks of gestation before (upper) and after (lower) excluding the implausible data in 2012.** The upper one shows the frequency distributions of birth weight before excluding. The lower one shows the frequency distributions of birth weight after excluding.
(TIF)
