# Supplementary figures and images for: Changes in Birth Weight between 2002 and 2012 in Guangzhou, China
Source: PLoS One. 2014 Dec 22;9(12):e115703. doi: 10.1371/journal.pone.0115703 (PMC4274089; doi:10.1371/journal.pone.0115703)

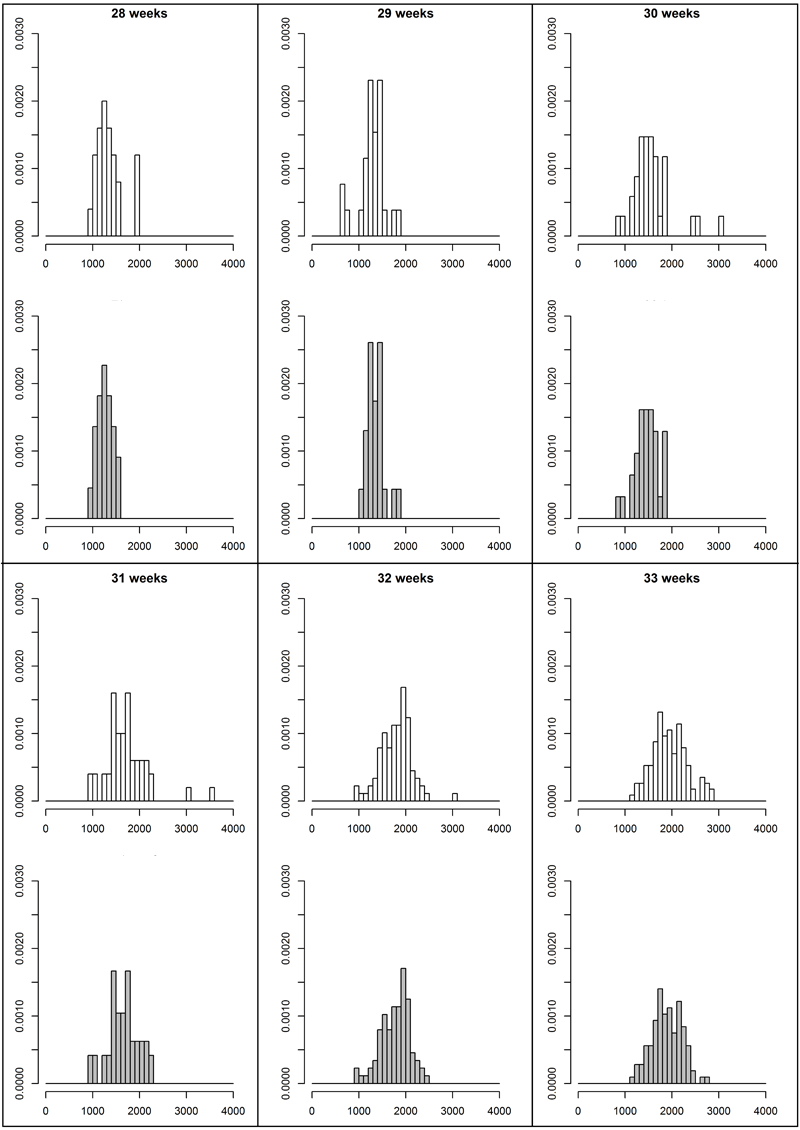

Supplement: S1 Fig — Frequency distributions of birth weight for 28-33 weeks of gestation before (upper) and after (lower) excluding the implausible data in 2002. The upper one shows the frequency distributions of birth weight before excluding. The lower one shows the frequency distributions of birth weight after excluding. (TIF) [file pone.0115703.s001.tif]

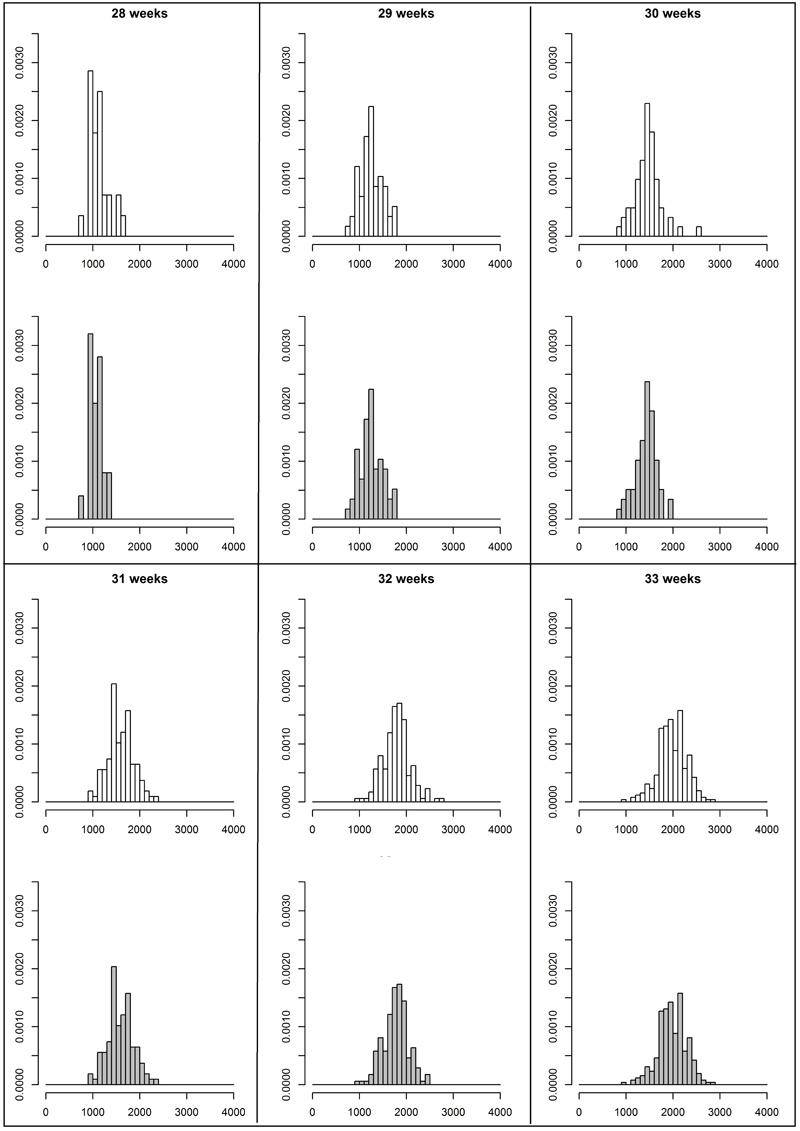

Supplement: S2 Fig — Frequency distributions of birth weight for 28-33 weeks of gestation before (upper) and after (lower) excluding the implausible data in 2012. The upper one shows the frequency distributions of birth weight before excluding. The lower one shows the frequency distributions of birth weight after excluding. (TIF) [file pone.0115703.s002.tif]
